# Single-cell transcriptomics reveals that tenocyte-derived MIF contributes to tendon injury pathogenesis

**DOI:** 10.1016/j.isci.2026.117411

**Published:** 2026-09-01

**Authors:** Cheuk Hin Kot, Xiaojie Xu, Peifeng Hong, Xu Liang, Gerun Chen, Zebin Ma

**Affiliations:** 1 Department of Orthopaedics, The First Affiliated Hospital of Shantou University Medical College, Shantou, Guangdong, China; 2 School of Biomedical Sciences, The Chinese University of Hong Kong, Hong Kong, China; 3 Department of Orthopaedics, Zhuhai Hospital of Integrated Traditional Chinese and Western Medicine (Zhuhai Hospital Affiliated to Faculty of Chinese Medicine, Macau University of Science and Technology), Zhuhai, Guangdong, China

**Keywords:** tendon injury, single-cell transcriptomics, MIF, osteogenesis

## Abstract

Tendon injury is accompanied by inflammation and pathological tissue remodeling, yet the underlying cellular and molecular mechanisms remain incompletely understood. Reanalysis of single-cell transcriptomic data across tendon-healing time points revealed dynamic cellular heterogeneity, including eight transcriptionally distinct mesenchymal subpopulations and eight immune-cell clusters. CellChat predicted relatively strong outgoing signaling from mesenchymal populations and incoming signaling to myeloid populations during repair. MIF-associated communication between selected tenocyte and myeloid populations was predicted to increase during the acute injury stage, involving the MIF-(CD74+CXCR4) and MIF-ACKR3 ligand-receptor pairs. MIF expression increased in injured tendon and was associated with inflammatory, apoptotic, stress-related, and histopathological changes. Pharmacological treatment with 4-IPP delivered in a photocrosslinked F127DA hydrogel formulation (F127@4-IPP) attenuated inflammation and osteogenic changes and improved histological outcomes in the mouse injury model. These findings support further evaluation of MIF-associated signaling and F127@4-IPP intervention in tendon injury.

## Highlights

• scRNA-seq reanalysis identified mesenchymal-myeloid communications during tendon injury
• MIF-associated signaling was predicted to increase during the acute injury stage
• F127@4-IPP attenuated histopathological and osteogenic changes in the mouse injury model

## Graphical abstract

**Figure:**
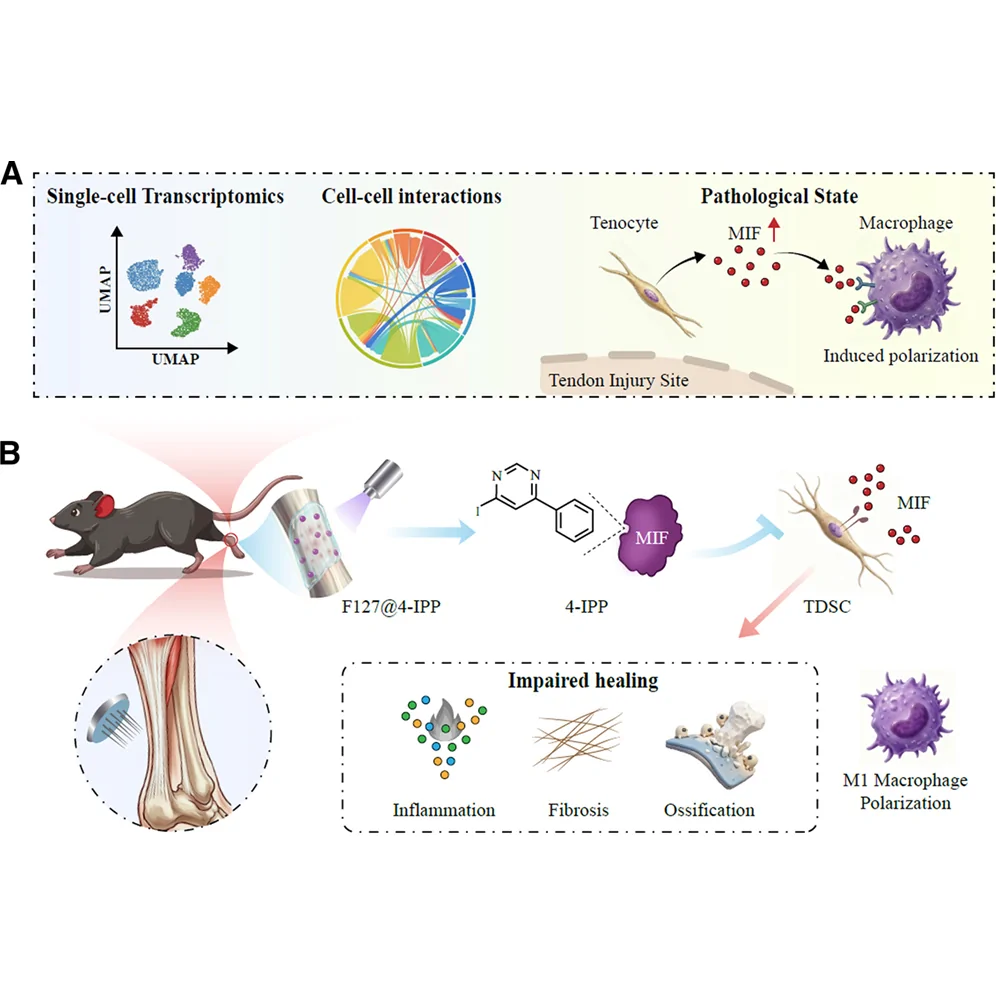

## Teaser

Health sciences

## Introduction

Tendons are dense fibrous connective tissues that serve as force transducers, which connect skeletal muscle to bone and facilitate movement and joint stability.^1^ With their tensile strength, tendons transmit the mechanical loading force generated by muscle contraction to the bone, which enables precise control of locomotion while storing and releasing elastic energy to enhance movement efficiency.^2^^,^^3^

Tendinopathies represent one of the most prevalent musculoskeletal disorders, accounting for approximately 30%–50% of all sports-related injuries and affecting millions of individuals worldwide annually.^4^ These degenerative conditions, characterized by activity-related pain, swelling, and functional impairment, significantly compromise mobility and quality of life across athletic and general populations.^5^ Despite the substantial socioeconomic burden, current therapeutic interventions remain palliative rather than curative, offering only short-term symptomatic relief without addressing underlying pathological mechanisms.^6^ The absence of disease-modifying treatments highlights critical knowledge gaps in tendinopathy pathogenesis and underscores the need for improved molecular understanding to develop targeted therapeutic strategies.

With the rapid advancement of transcriptomic technologies, including bulk RNA sequencing and single-cell RNA sequencing (scRNA-seq), it has become possible to examine gene expression across diverse cell populations with much greater resolution, thereby providing new insight into the cellular heterogeneity that underlies tendinopathy progression.^7^ In particular, Huang et al. constructed both a single-cell atlas and bulk transcriptomic gene expression profile of puncture-induced tendon injury at multiple time points, offering a dynamic view of the molecular and cellular landscape during tendon repair and degeneration.^8^ Their study identified mesenchymal cells and macrophage populations as important contributors to the pathological process, suggesting that these cell types play roles in shaping disease progression. However, this may still be insufficient to fully characterize changes in the tendon microenvironment, especially when temporal interactions and crosstalk between distinct cell populations have not been thoroughly examined. Tendon injury is not only a consequence of intrinsic alterations within a single cell type but also the result of complex intercellular communication among stromal, immune, and resident tissue cells over time.^9^ Previous studies have further indicated that myeloid populations serve as central regulators of the inflammatory response during tendon pathology, whereas the active contribution of mesenchymal cells to the modulation of the immune microenvironment has been relatively overlooked.^10^^,^^11^

In this study, we reanalyzed the published dataset (GEO: GSE288444) to investigate functional heterogeneity and predicted intercellular communication during tendon healing. Building on the cellular and temporal framework established by Huang et al., we focused on communication between mesenchymal and myeloid populations and identified macrophage migration inhibitory factor (MIF) as a candidate signaling pathway associated with the acute injury stage. We subsequently evaluated the association of MIF with inflammatory, fibrotic, and osteogenic changes using microarray data of human tendon samples, mouse models, and *in vitro* experiments. Finally, we examined the therapeutic potential of pharmacological MIF inhibition using an F127@4-IPP delivery system.

## Results

### scRNA-seq revealed the dynamic changes in cellular populations following puncture injury in tendon

**Figure 1:**
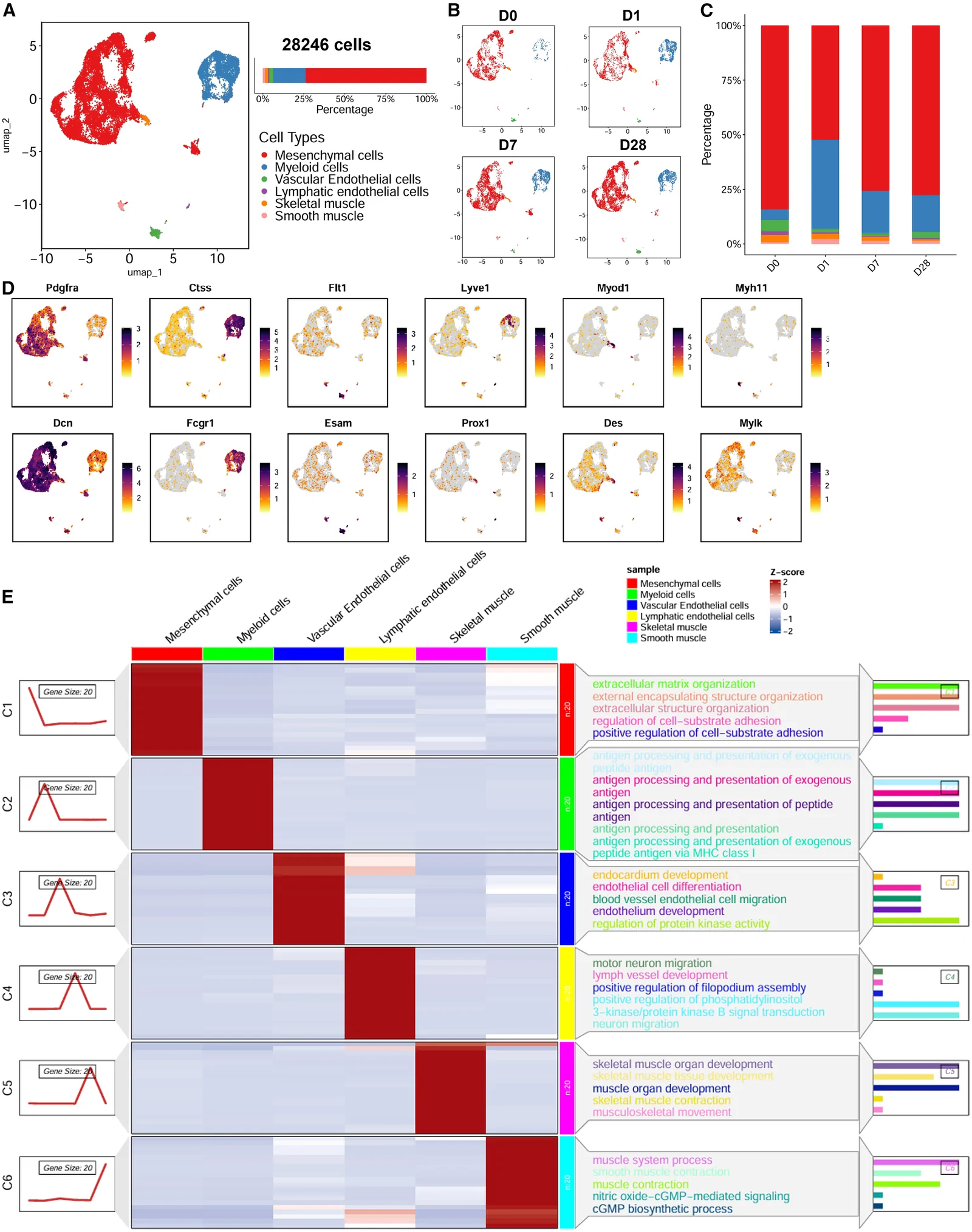
scRNA-seq analysis reveals the dynamic changes in the microenvironment during tendon healing after puncture-induced injury (A) The UMAP plots showing the 6 distinct cell types identified. (B) The UMAP plots showing the cell populations at different time points. (C) The compositions of different cell types at different time points. (D) The feature plots showing the expression of the indicated markers for each cell type. (E) The heatmap displays the top gene sets and top 5 GO pathways for each cell type. The data were sourced from GEO: GSE288444.

To elucidate the cellular heterogeneity across distinct temporal phases post-puncture injury, we reanalyzed scRNA-seq datasets (GEO: GSE288444).^8^ Following the exclusion of low-quality cells exhibiting elevated mitochondrial gene content and diminished feature counts, along with doublet removal, we retained 28,246 cells. Unsupervised clustering resolved six transcriptionally distinct cellular compartments (Figure 1A). Mesenchymal populations were characterized by elevated expression of markers *Pdgfra* and *Dcn*, with concomitant enrichment of Gene Ontology (GO) terms associated with extracellular matrix organization and structural assembly^12^ (Figures 1D and 1E). Myeloid lineages displayed high expression of *Ctss* and *Fcgr1*, consistent with functional specialization in antigen processing and presentation.^13^ Vascular endothelial cells exhibited pronounced expression of *Flt1* and *Esam*, aligned with endothelial differentiation programs.^14^ Lymphatic endothelial cells were defined by high *Lyve1* and *Prox1* expression, which are consistent with their involvement in lymphangiogenesis.^15^ Skeletal muscle cells expressed *Myod1* and *Des*, which are enriched for myogenic developmental and contractile processes.^16^ Smooth muscle compartments were characterized by *Myh11* and *Mylk* expression, which are associated with smooth muscle contraction.^17^

A substantial expansion of the myeloid lineage was observed acutely post-injury and reached its peak level at day 1 post-injury (D1), followed by a progressive decline through D28 (Figures 1B and 1C). In contrast, mesenchymal cell populations demonstrated a significant drop, reaching the lowest levels at D1 prior to a gradual reconstitution by D28. Concomitantly, both lymphatic and vascular endothelial compartments exhibited marked depletion at D1, and they sustained at attenuated levels throughout the reparative timeline.

### Transcriptomic stratification of mesenchymal cell subpopulations

**Figure 2:**
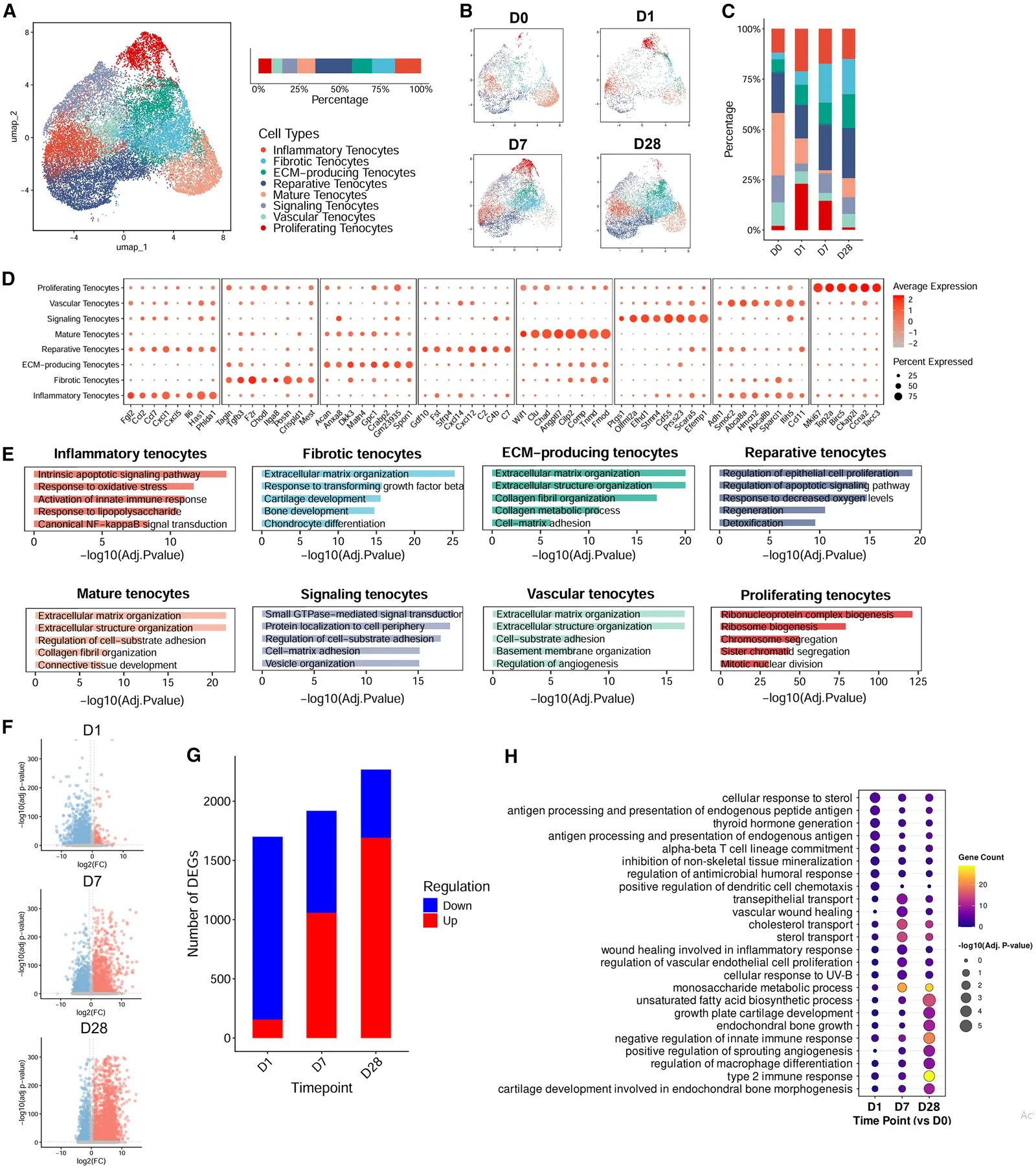
Cellular characterization of mesenchymal cell subtypes from scRNA-seq (A) The UMAP plots showing the 8 distinct mesenchymal cell subtypes identified. (B) The UMAP plots showing mesenchymal cell populations at different time points. (C) The compositions of different subtypes at different time points. (D) The dot plots showing the expression of specific markers for each subtype. (E) GO pathway enrichment analysis of the mesenchymal cell subtypes. (F) Volcano plots for differentially expressed genes (DEGs) at D1, D7, and D28, respectively, compared to D0. (G) Bar chart showing the quantification of upregulated and downregulated DEGs at D1, D7, and D28. (H) GO enrichment analysis of upregulated pathways at different time points.

Given the dominant role of mesenchymal cells in tendon homeostasis and collagen fibrillogenesis, we performed subclustering analysis, which delineated eight transcriptionally distinct mesenchymal subsets, including inflammatory, fibrotic, extracellular matrix (ECM)-producing, reparative, mature, signaling, vascular, and proliferating tenocytes^18^ (Figure 2A). Inflammatory tenocytes demonstrated upregulation of pro-inflammatory mediators *Cxcl1*, *Ccl2*, *Ccl7*, and *Il6*, with enrichment for innate immune activation and oxidative stress response pathways^19^ (Figures 2D and 2E). Fibrotic tenocytes exhibited elevated expression of *Postn*, *Tagln*, and *Tgfb3*, and they were enriched for extracellular matrix organization, chondrogenesis, and osteogenic differentiation.^20^ ECM-producing tenocytes showed high expression of *Matn4* and *Gpc1*, implicated in ECM assembly, and were enriched for collagen fibril organization.^21^^,^^22^ Reparative tenocytes expressed *Cxcl12* and *Cxcl14*, alongside complement components *C2*, *C4b*, and *C7*, which facilitate debridement of necrotic debris.^23^ This subset was further characterized by enrichment for hypoxia response, detoxification, and regenerative pathways. Mature tenocytes expressed definitive tenogenic markers Tnmd and Fmod, with concomitant enrichment for connective tissue development.^24^ Inflammatory and proliferating tenocyte subsets underwent a transient expansion, peaking at D1 and subsequently diminishing by D28 (Figures 2B and 2C). Fibrotic tenocytes exhibited progressive expansion and attained maximal representation at D28. Mature tenocyte populations declined initially, reaching a trough at D7, followed by reconstitution at D28. These findings indicated the transition from an inflammatory and reparative phenotype in the early post-injury phase to a fibrotic state by D28, driving tissue remodeling and impaired healing.

### Deconvolution of myeloid cell heterogeneity

**Figure 3:**
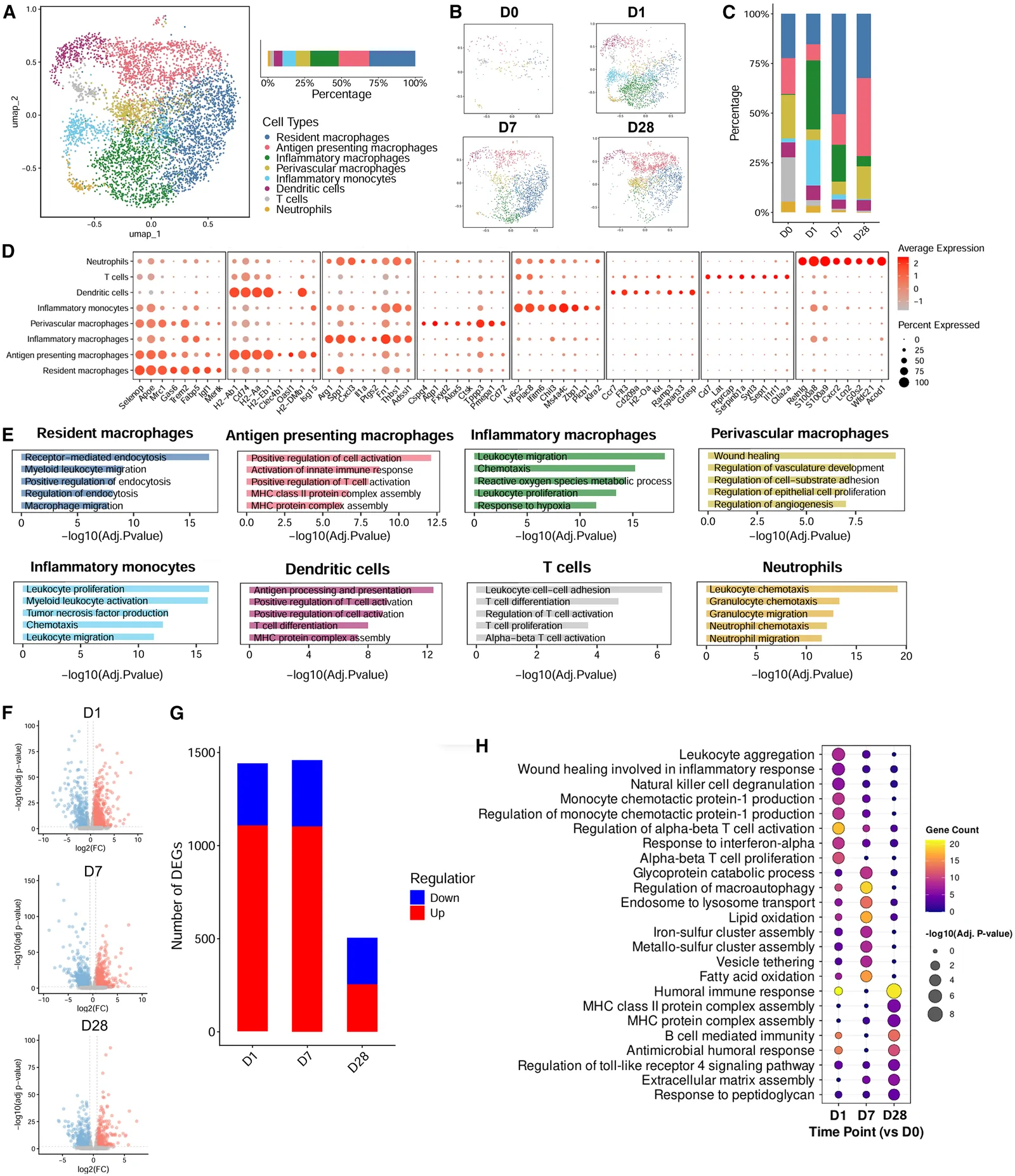
Cellular characterization of myeloid cell subtypes from scRNA-seq (A) The UMAP plots showing 8 distinct myeloid cell subtypes identified. (B) The UMAP plots showing myeloid cell populations at different time points. (C) The compositions of different subtypes at different time points. (D) The dot plots showing the expression of specific markers for each subtype. (E) GO pathway enrichment analysis of the myeloid cell subtypes. (F) Volcano plots for DEGs at D1, D7, and D28, respectively, compared to D0. (G) Bar chart showing the quantification of upregulated and downregulated DEGs at D1, D7, and D28. (H) GO enrichment analysis of upregulated pathways at different time points.

In light of the established regulatory role of myeloid cells in inflammatory cascades and disease pathogenesis, we conducted subclustering of the myeloid compartment, resolving eight distinct subsets, including resident macrophages, antigen-presenting macrophages, inflammatory macrophages, perivascular macrophages, inflammatory monocytes, dendritic cells, T cells, and neutrophils^25^ (Figure 3A). Resident macrophages were characterized by expression of *Gas6*, *Trem2*, *Fabp5*, and *Mertk*, with enrichment for endocytic regulation^4^^,^^26^ (Figures 3D and 3E). Antigen-presenting macrophages demonstrated elevated expression of MHC class II constituents (*H2-Ab1*, *H2-Aa*, *H2-Eb1*, *H2-DMb1*) and enrichment for adaptive immune activation.^27^ Inflammatory macrophages exhibited expression of *Spp1*, *Cxcl3*, *Il1a*, and *Ptgs2*, alongside enrichment for leukocyte chemotaxis and hypoxia response.^27^ Inflammatory monocytes expressed *Ly6c2* and *Plac8*, concomitant with activation markers *Chil3*, *Zbp1*, and *Plcb1*, and were enriched for chemotactic and TNF-production pathways.^27^^,^^28^ A substantial influx of inflammatory macrophages and monocytes was observed at D1, indicating the injury-induced polarization of macrophages toward an M1 phenotype and recruitment of circulating monocytes (Figures 3B and 3C). Resident, antigen-presenting, and perivascular macrophage populations contracted at D1 but demonstrated substantial recovery after D7. These data indicated the temporal shift from an early pro-inflammatory influx of M1-like macrophages and monocytes to the subsequent restoration of resident subsets, highlighting the resolution of acute inflammation.

### Temporally resolved functional profiling of mesenchymal and myeloid compartments

While bulk transcriptomic analyses from previous studies have delineated differentially expressed genes (DEGs) and associated biological processes during tendon healing, the cell type-specific contributions remain incompletely elucidated. We identified mesenchymal cell DEGs at successive time points relative to uninjured controls (Figure 2F). The early responses at D1 were dominated by transcriptional downregulation, whereas late responses at D28 were characterized by upregulation, with the overall magnitude of perturbation increasing over time (Figure 2G). DEGs at D1 were enriched for T cell lineage commitment, antimicrobial humoral responses, and dendritic cell chemotaxis (Figure 2H). D7 DEGs were enriched for wound healing, sterol transport, and monosaccharide metabolism, suggesting a transition toward reparative and metabolic reprogramming. D28 DEGs were enriched for chondrogenic differentiation, angiogenic sprouting, and type 2 immunity, indicative of late-stage matrix remodeling and immunoregulatory activity.

We subsequently interrogated the myeloid cell populations, a principal driver of inflammatory responses. We identified myeloid cell DEGs at different time points compared to uninjured controls (Figure 3F). Transcriptional profiling revealed a predominance of upregulated over downregulated DEGs at D1 and D7 compared to baseline, with levels returning to equilibrium by D28 (Figure 3G). Myeloid cell DEGs at D1 were enriched for leukocyte aggregation, natural killer cell degranulation, monocyte chemotaxis, and T cell activation (Figure 3H). D7 DEGs were enriched for glycoprotein catabolism, macroautophagy, and lipid oxidation. D28 DEGs were enriched for humoral immunity, MHC complex assembly, TLR4 signaling, ECM assembly, and peptidoglycan response.

### Global and mesenchymal/myeloid cell signaling pathways after injury

**Figure 4:**
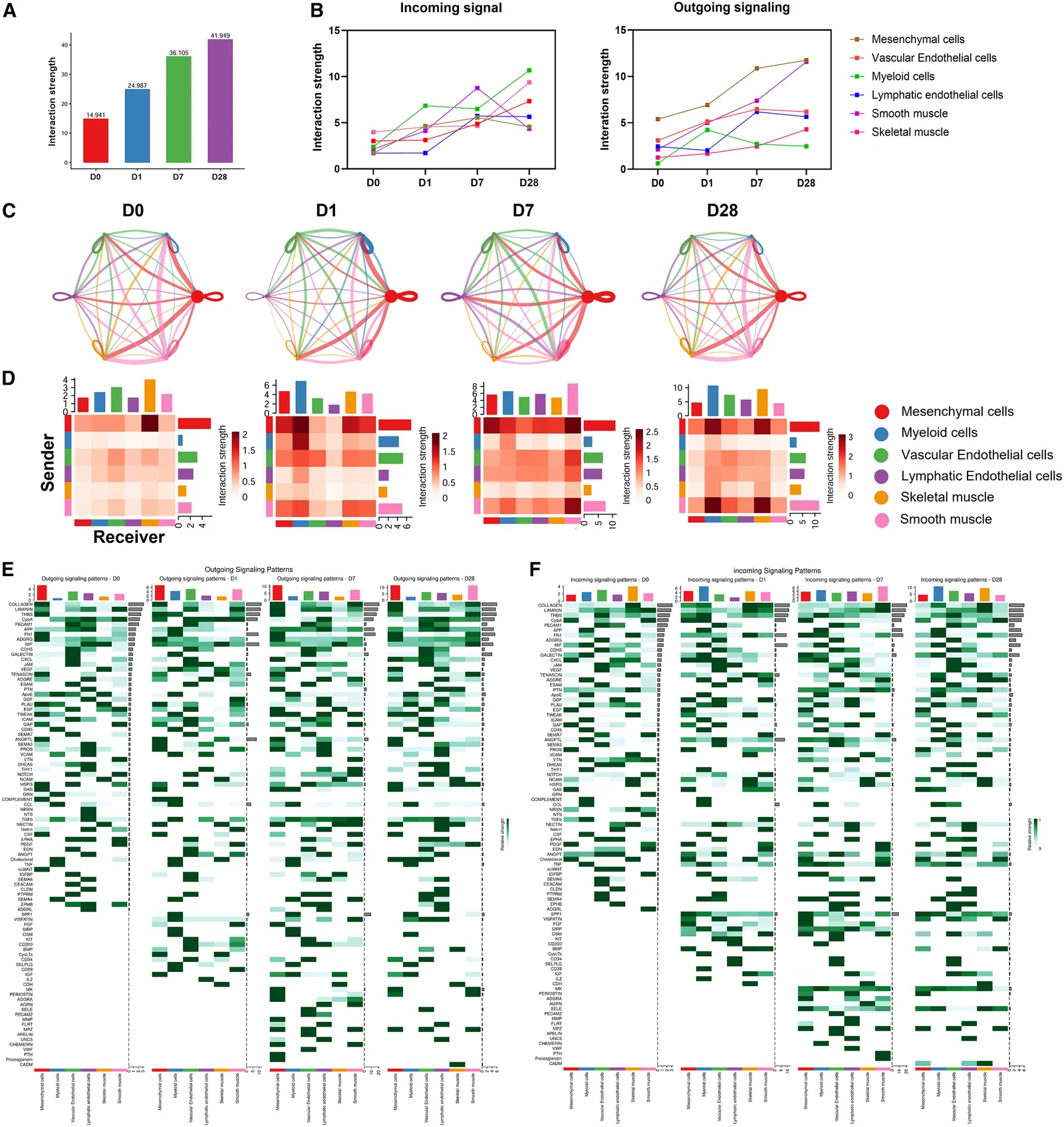
Cell-cell interaction network during tendon healing after puncture-induced injury (A) The interaction strength among all cell populations in different time points. (B) The interaction strength of signaling pathways incoming from or outgoing from different cell populations. (C) Circle plots and heatmap representing the cell pairs for the interaction strengths of signaling pathways. (D) Relative strength of outgoing and incoming signaling pathways at different time points. The bar chart at the top shows the interaction strength of each cell type as signal senders or receivers. Bar chart at the right shows the strength of each signaling pathway. (E and F) Relative strengths of outgoing and incoming signaling pathways in major populations at D0, D1, D7, and D28. Bar graphs at the top of each heatmap show cumulative signaling strengths per cell population. Bar graphs at the right of each heatmap show cumulative signaling strength per pathway.

To investigate cell-cell communication across cell types in tendon tissue post-injury, we employed CellChat analysis, which revealed a gradual increase in interaction strength from the uninjured state to D28^29^ (Figure 4A). To identify key cell types within the complex signaling network, we categorized interactions as incoming or outgoing for each cell type (Figures 4B, 4E, and 4F). The myeloid cell population exhibited elevated cumulative strength of incoming pathway signals as early as D1 post-injury. Notably, in the inferred CellChat network, mesenchymal cells exhibited the highest cumulative outgoing signaling strength among the analyzed cell populations, whereas myeloid cells showed relatively high incoming signaling strength. Moreover, the cumulative strength of incoming signals to mesenchymal cells doubled from D0 to D28, relative to other cell types. Mapping of ligand-receptor interactions predicted that cell-cell interactions between mesenchymal cells and myeloid cells were highest at D1 (Figures 4C and 4D). Mesenchymal cells also engaged in autocrine signaling and crosstalk with smooth muscle at D7, while high communication strength was observed between mesenchymal cells and myeloid cells, and with skeletal muscle at D28. These findings imply that mesenchymal cells and myeloid cells exhibit elevated and dynamic signaling activities during the healing process following tendon injury.

**Figure 5:**
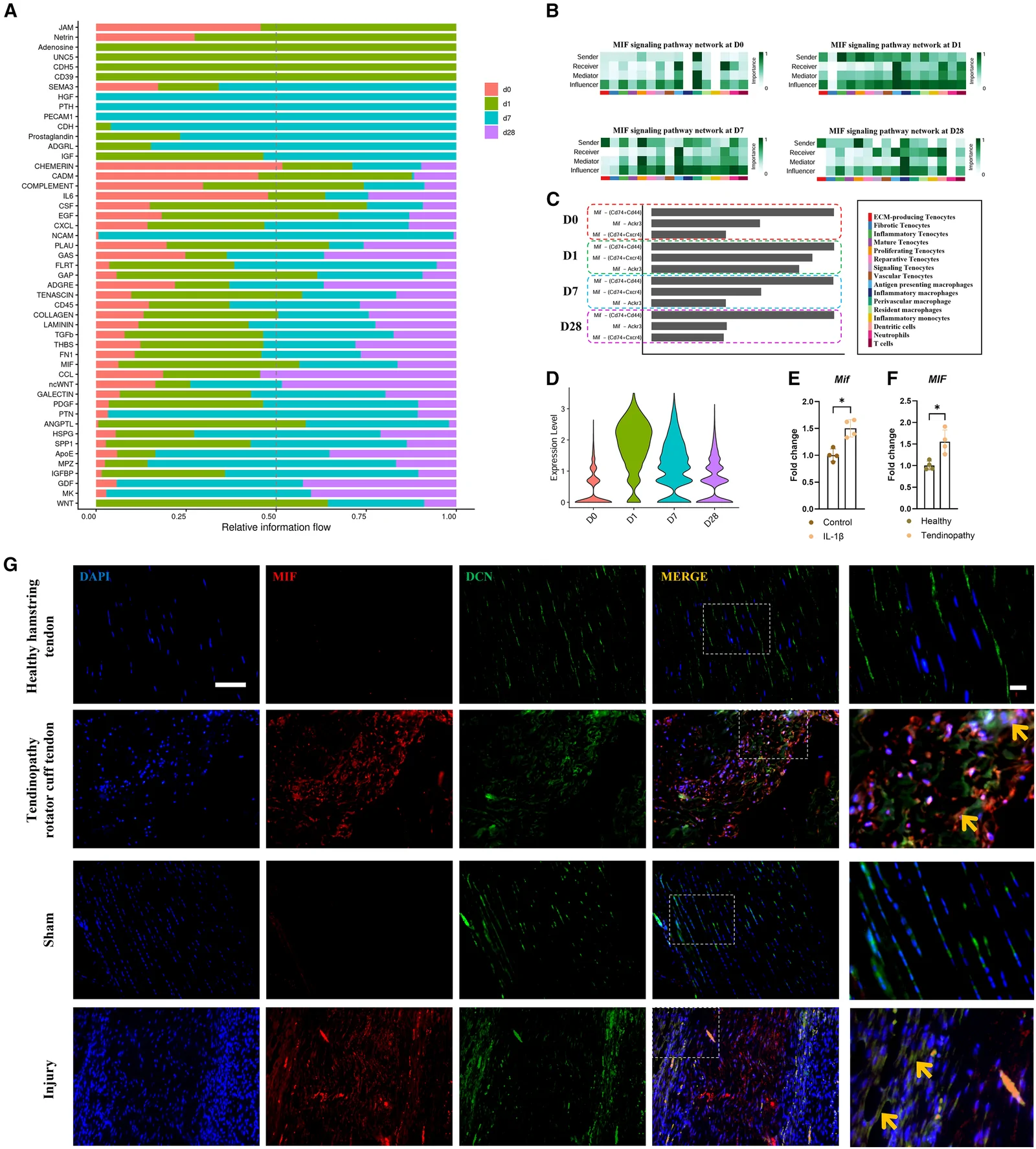
MIF-associated signaling and expression after tendon injury (A) Relative information flow for predicted signaling pathways at different time points. (B) Heatmap showing predicted network centrality in mesenchymal and myeloid subpopulations. White indicates low relative centrality, and green indicates high relative centrality. (C) Relative contributions of MIF-associated ligand-receptor pairs at different time points. (D) Violin plots showing *Mif* expression across time points. (E) Relative *Mif* mRNA expression in mouse TDSCs with or without IL-1 treatment (*n* = 4 per group). (F) Relative *Mif* mRNA expression in TDSCs isolated from healthy hamstring and tendinopathic rotator cuff samples (*n* = 4 per group). (G) Representative immunofluorescence images showing MIF and DCN staining in healthy hamstring and tendinopathic rotator cuff samples and in mouse tendons from the sham and injury groups. DAPI labels nuclei. Dashed boxes indicate the enlarged regions, and yellow arrows indicate overlapping MIF and DCN signals. Scale bars, 50 μm (original images) and 10 μm (magnified images). Statistical significance was assessed using an unpaired two-tailed Student’s *t* test. Data are shown as mean ± SD; ∗*p* < 0.05.

CellChat predicted 49 potentially active signaling pathways involving mesenchymal and myeloid populations (Figure 5A). At D1, the acute inflammatory response was characterized by a robust upregulation of pathways, including MIF and CSF, which peaked at this early time point.^30^^,^^31^ This phase was further defined by the specific activation of CD39 and adenosine signaling, pathways critically implicated in the inflammatory response.^32^^,^^33^ By D7, signaling dynamics shifted toward the regenerative phase, marked by the emergence of growth factor pathways such as HGF and PTH.^34^^,^^35^ Moreover, angiogenesis-related pathways, including PECAM1 and CDH, were uniquely activated at D7.^36^ This period also showed the upregulated pathways governing ECM deposition and organization, notably SPP1, NCAM, and SEMA3.^36^^,^^37^^,^^38^ Finally, at D28, the GAS signaling reached its peak, a pathway associated with fibrotic remodeling.^39^ Non-canonical WNT (ncWNT) signaling showed the same pattern and is known to suppress tenogenic differentiation and promote pathological matrix accumulation.^40^

### MIF pathway upregulation in the acute injury stage

CellChat predicted that mesenchymal populations exhibited relatively high outgoing MIF-associated signaling, whereas myeloid populations exhibited relatively high incoming signaling during the early injury stage.^30^ Under homeostatic conditions, perivascular macrophages were predicted to be the major signal senders, whereas antigen-presenting macrophages and dendritic cells were the principal receivers (Figure 5B). At D1 after injury, predicted MIF-associated signaling expanded to include mesenchymal subclusters, with inflammatory and proliferating tenocytes showing relatively high outgoing signaling. At D7, ECM-producing and proliferating tenocytes were predicted to be the principal senders, whereas antigen-presenting macrophages were the major receivers. By D28, predicted MIF-associated signaling within mesenchymal populations was reduced; ECM-producing and proliferating tenocytes remained the principal senders, whereas dendritic cells and antigen-presenting macrophages were the principal receivers. CellChat also predicted increased communication probabilities for the MIF-(CD74+CXCR4) and MIF-ACKR3 ligand-receptor pairs at D1, followed by lower predicted probabilities at D7 and D28 (Figure 5C).

We next examined MIF signaling across time points in the puncture-induced injury model. *Mif* expression increased sharply at D1 and declined at D7 and D28. Immunohistochemical staining confirmed increased MIF protein expression at D1, with levels returning toward those of the sham group by D7–D28 (Figure 5D). *In vitro* qPCR analysis showed increased *Mif* mRNA expression in tendon-derived stem cells (TDSCs) treated with IL-1 (Figure 5E). MIF expression was higher in TDSCs derived from tendinopathic rotator cuff samples (mean age: 53.50 ± 3.51 years) than in those derived from healthy hamstring samples (mean age 34.75 ± 5.91 years) (Figure 5F). Immunofluorescence staining showed visually greater overlapping MIF/DCN signals in tendinopathic rotator cuff samples (Figure 5G). We further examined MIF expression *in vivo* using a puncture-induced Achilles tendon injury model (Figure S2). Injured tendons showed greater overlapping MIF/DCN signals than sham tendons (Figure 5G). Collectively, these findings support increased MIF expression under the examined tendon injury conditions and suggest that tenocyte-lineage cells may represent one cellular source of MIF.

### Increased MIF activity is associated with tendon pathological changes

**Figure 6:**
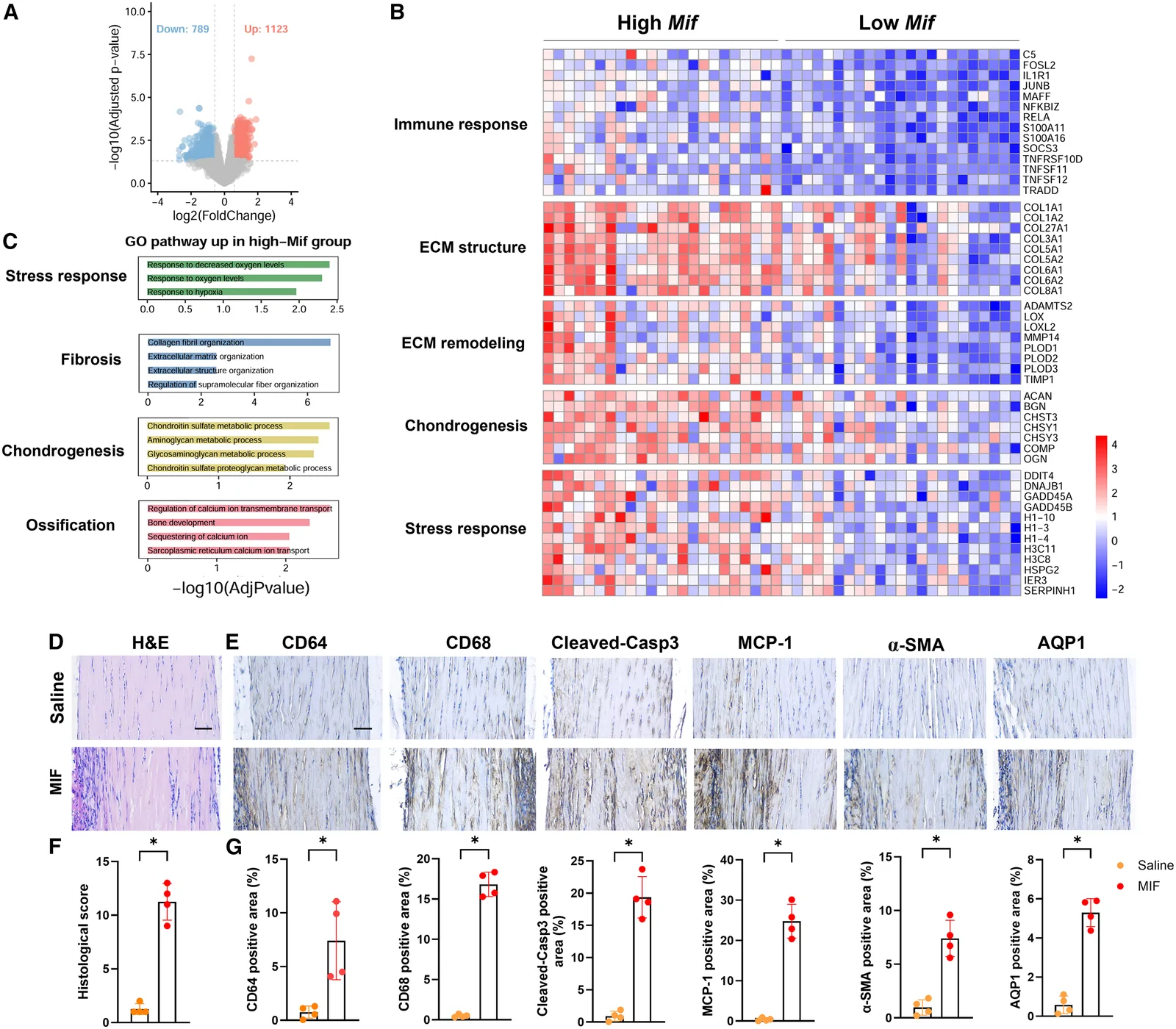
MIF-associated molecular signatures and pathological changes in tendon (A) Volcano plots showing DEGs between high- and low-*MIF* groups in GSE26051. (B) Heatmap showing selected genes in the high- and low-*MIF* groups. Blue and red indicate relatively low and high expression, respectively. (C) Representative GO terms enriched among genes upregulated in the high-*MIF* group. (D) Representative H&E-stained sections at 1 week. (E) Representative immunohistochemical staining for CD64, CD68, Cleaved-Casp-3, MCP-1, α-SMA, and AQP1 at 1 week. (F) Histological scores (*n* = 4 per group). (G) Semi-quantitative analysis of marker staining (*n* = 4 per group). Statistical significance was assessed using an unpaired two-tailed Student’s *t* test. Data are shown as mean ± SD; ∗*p* < 0.05.

To examine the clinical context of MIF expression, we analyzed a microarray dataset of tendon samples (GSE26051), ranked MIF expression among 46 patients, and divided the samples into high- and low-expression groups using the median MIF value.^41^ In the low-*MIF* expression group, there were 789 genes downregulated and 1,123 genes upregulated (Figure 6A). We observed that the high *MIF* expression group exhibited a greater degree of degenerative progression, as evidenced by the upregulation of immune response genes (e.g., *S100A11*, *S100A16*, and TNF family), ECM-related genes (collagen family, *ADAMTS2*, *MMP14*, and *TIMP1*), chondrogenic genes (*ACAN* and *BGN*), and cellular stress-related genes (*DDIT4* and *DNAJB1*) (Figure 6B). GO enrichment analysis also revealed the upregulation of response to hypoxia, collagen fibril organization, chondroitin sulfate metabolic process, and bone development (Figure 6C). These associations suggest that high MIF expression accompanies molecular programs related to cellular stress, inflammation, fibrosis, and chondrogenic or osteogenic remodeling.

To further examine the effects associated with increased local MIF exposure, recombinant mouse MIF protein was injected around the Achilles tendons of mice. This treatment resulted in cellular infiltration, inflammation, and disrupted collagen alignment at week 1 (Figures 6D and 6F). Immunohistochemical analysis revealed upregulated protein expression of inflammatory marker MCP-1, apoptotic marker Cleaved-Casp3, fibrotic marker α-SMA, and migration-regulating marker AQP1 (Figures 6E and 6G). We also observed the elevated expression of CD64 and CD68 markers, reflecting an increasing number of macrophages and a higher tendency toward M1 polarization (Figures 6E and 6G).

### 4-IPP modulates pathological responses in TDSCs and the inflammatory effects of TDSC-conditioned medium

**Figure 7:**
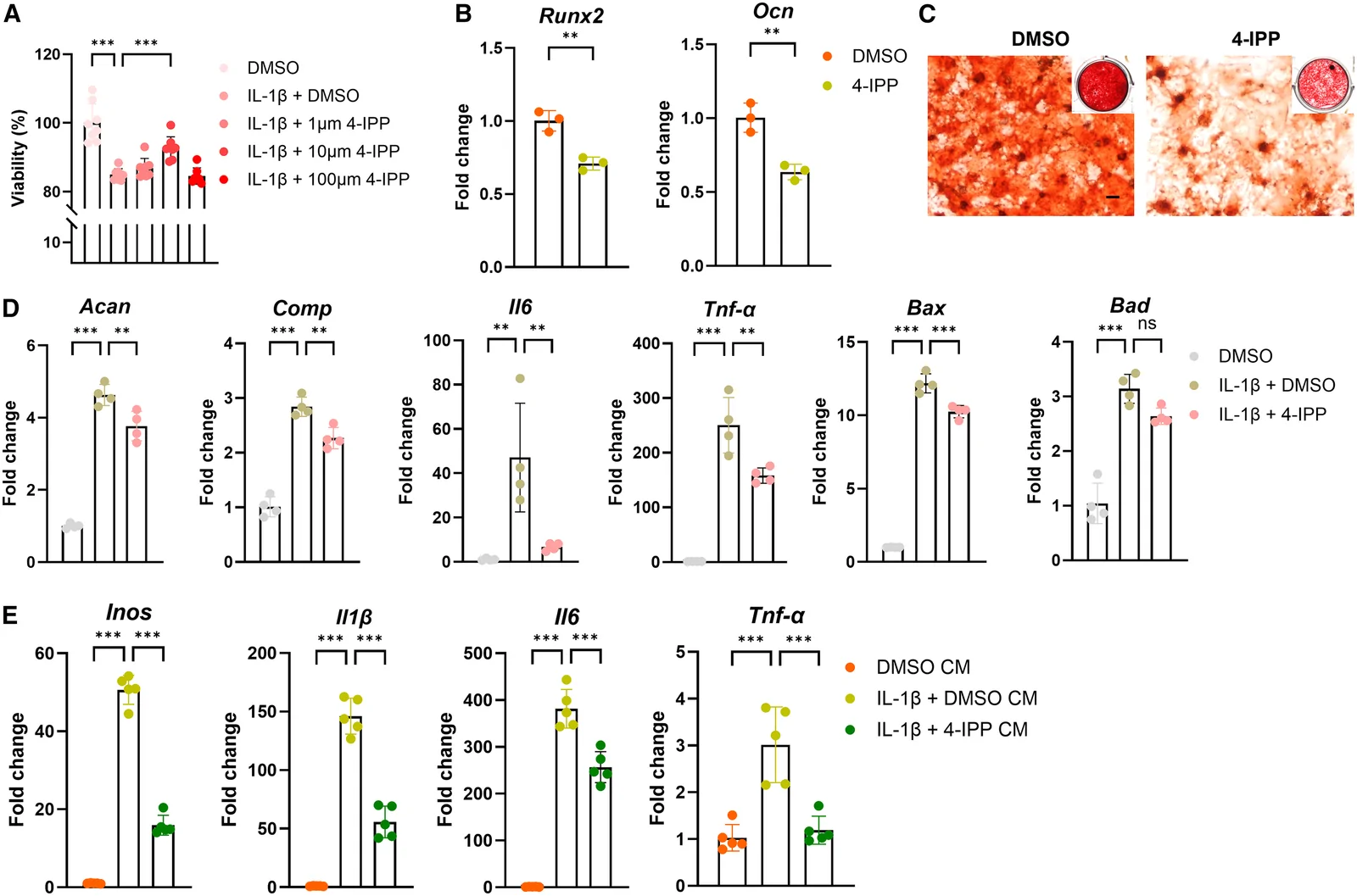
MIF inhibitor 4-IPP treatment ameliorates pathogenesis of TDSCs and macrophages (A) Cell viability of 4-IPP treated TDSCs after IL-1β stimulation (*n* = 7–8 per group). (B) mRNA expression of osteogenic markers *Runx2* and *Ocn* in TDSCs from different groups (*n* = 3 per group). (C) Representative images showing the ARS staining of TDSCs under osteogenic medium on day 21 with or without 4-IPP treatment. Scale bars, 50 μm. (D) mRNA expression of inflammatory markers *Il6* and *Tnf-a*, apoptotic markers *Bax* and *Bad*, and chondrogenic markers *Acan* and *Comp* in TDSCs from different groups (*n* = 5 per group). (E) Relative mRNA expression of *Inos*, *Il1β*, *Il6*, and *Tnf-α* in RAW264.7 cells following exposure to conditioned medium collected from the indicated TDSC treatment groups (*n* = 5 per group) (*n* = 5 per group). Statistical significance was calculated using one-way ANOVA with Tukey test; data are shown as mean ± SD. ∗∗*p* < 0.01, ∗∗∗*p* < 0.001.

To further investigate the effects of MIF suppression on TDSC function *in vitro*, we treated mouse Achilles tendon-derived TDSCs with the MIF inhibitor, 4-IPP.^42^ CCK8 assay confirmed increased viability of IL-1β-stimulated TDSCs treated with 10 μM 4-IPP, and this concentration was used for subsequent *in vitro* studies (Figure 7A). Furthermore, 4-IPP treatment reversed the upregulation of inflammatory (*Il6* and *Tnf-α*), apoptotic (*Bax* and *Bad*), and chondrogenic (*Acan* and *Comp*) markers in IL-1β–stimulated TDSCs (Figure 7D). We also observed that 4-IPP treatment reduced the expression of osteogenic markers (Runx2 and Ocn) and suppressed osteogenic differentiation, as assessed by Alizarin Red S staining under osteogenic induction (Figures 7B and 7C).

Because CellChat predicted relatively high incoming MIF-associated signaling in myeloid populations during acute injury, we next examined whether conditioned medium from 4-IPP-treated TDSCs altered inflammatory gene expression in RAW264.7 macrophage-like cells. Conditioned media were collected from TDSCs treated with IL-1β alone or IL-1β plus 4-IPP, then applied to RAW 264.7 macrophage cultures. Macrophages exposed to supernatant from IL-1β-stimulated TDSCs exhibited marked upregulation of inflammatory markers (*Inos*, *Il-1β*, *Il6*, and *Tnf-α*). In contrast, macrophages incubated with supernatant from TDSCs co-treated with IL-1β and 4-IPP showed a pronounced reduction in the expression of these markers (Figure 7E). These results indicate that pharmacological treatment of TDSCs with 4-IPP altered the effect of their conditioned medium on inflammatory gene expression in RAW264.7 cells.

### siRNA-mediated MIF knockdown suppresses osteogenic differentiation in TDSCs

To complement the pharmacological inhibition experiments and further establish the role of endogenous MIF, we performed siRNA-mediated knockdown of MIF in TDSCs. Transfection with MIF siRNA significantly reduced *Mif* mRNA expression compared to the scramble siRNA group (Figure S4A). Under osteogenic induction conditions, TDSCs treated with MIF siRNA exhibited significantly reduced mRNA expression of osteogenic markers *Runx2* and *Ocn* compared to the scramble siRNA group at day 21 (Figure S4B). These loss-of-function data corroborate the 4-IPP findings and demonstrate that knockdown of endogenous MIF suppresses osteogenic differentiation of TDSCs.

### Characterization of blank and 4-IPP-loaded F127DA hydrogels

To characterize the F127DA hydrogel as a drug delivery platform, blank F127DA and 4-IPP-loaded F127DA hydrogels (F127@4-IPP) were successfully fabricated. Scanning electron microscopy (SEM) analysis revealed that the incorporation of 4-IPP did not visibly alter the interconnected porous network of the F127DA hydrogel (Figure S3A). The release profile of 4-IPP exhibited an early rapid release followed by a sustained release phase, reaching 51% ± 2.7% by day 14 (Figure S3B). The swelling ratios of blank and 4-IPP-loaded hydrogels at equilibrium were 264% ± 18% and 255% ± 20%, respectively, with both formulations approaching equilibrium within 24 h (Figure S3C). Encapsulation efficiency was determined to be 94.9% ± 3.5% at a theoretical loading content of 2.5% (w/w) (Figure S3D). *In vitro* degradation showed a gradual weight loss over 14 days, with no significant difference between groups (Figure S3E). Collectively, these results confirm that F127DA hydrogels maintain favorable physicochemical properties after drug loading, supporting their suitability for *in vivo* evaluation.

### F127@4-IPP treatment attenuates histopathological changes after tendon injury

**Figure 8:**
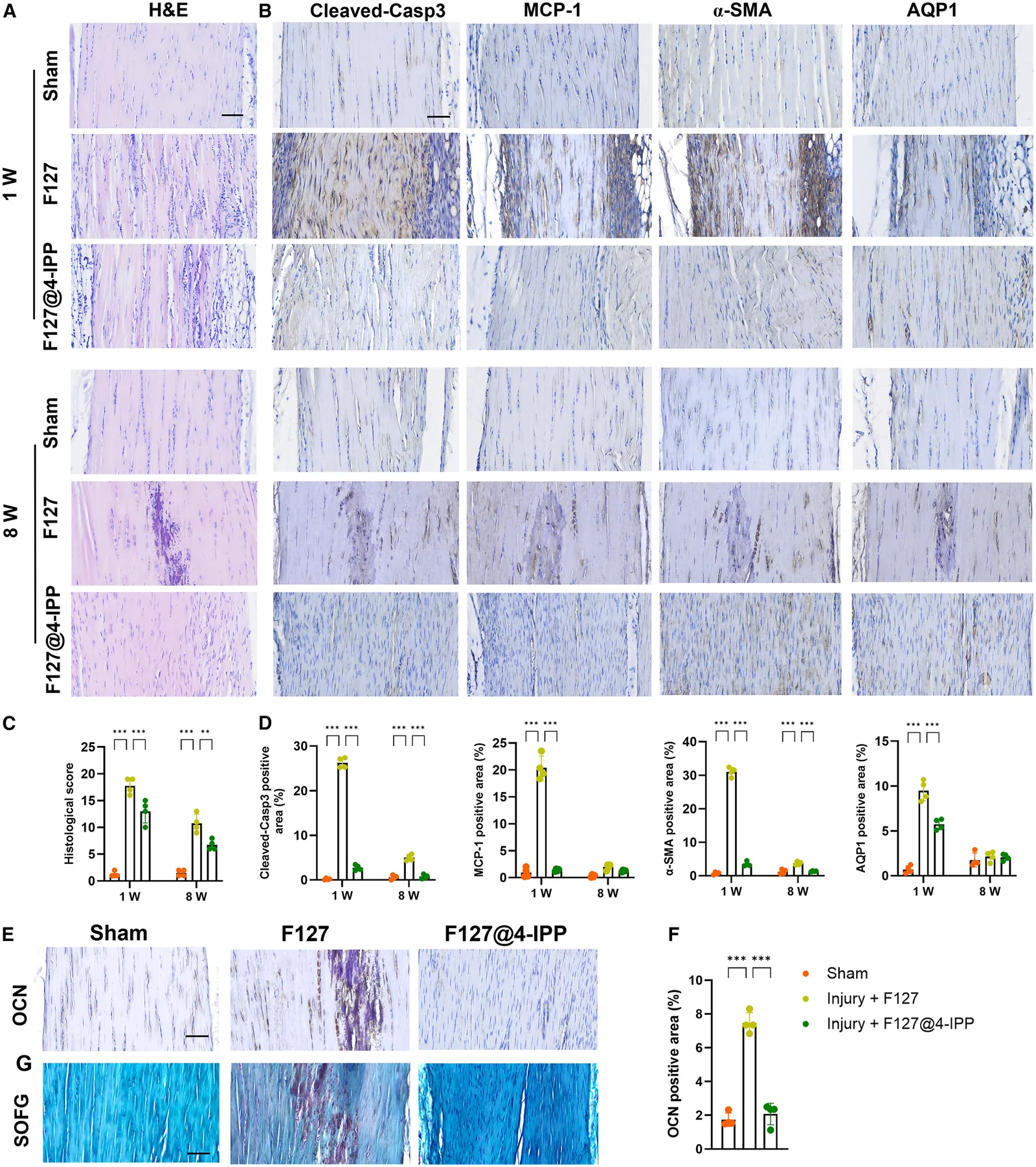
F127@4-IPP treatment attenuates histopathological and osteogenic changes after tendon injury (A) Representative H&E staining images from different groups at 1 and 8 weeks post-injury. Scale bars, 100 μm. (B) Representative IHC staining images for the markers Cleaved-Casp3, MCP-1, α-SMA, and AQP1 from different groups at 1 and 8 weeks post-injury. Scale bars, 100 μm. (C) Quantification of histological scores in different groups at 1 and 8 weeks post-injury (*n* = 4 per group). (D) Semi-quantitative analysis of Cleaved-Casp3, MCP-1, α-SMA, and AQP1 expression levels in different groups at 1 and 8 weeks post-injury (*n* = 4 per group). (E and F) Representative IHC staining images and semi-quantitative analysis for the markers *Ocn* in different groups at 8 weeks post-injury (*n* = 4 per group). Scale bars, 100 μm. (G) Representative SOFG staining images showing proteoglycan deposition in different groups at 8 weeks post-injury. Scale bars, 100 μm. Statistical significance was calculated using one-way ANOVA with Tukey test; data are shown as mean ± SD. ∗*p* < 0.05, ∗∗*p* < 0.01, ∗∗∗*p* < 0.001.

To investigate the effects of F127@4-IPP treatment on tendon healing, we employed a puncture-induced injury model and injected F127@4-IPP into the injury site. At 1-week post-injury, H&E staining revealed reduced hypercellularity, fewer small nucleated cells, and less apparent collagen disorganization in the F127@4-IPP group compared to the F127-alone group (Figure 8A). At 8 weeks post-injury, we observed the presence of rounded cells and bone formation in the F127 group, but not in the F127@4-IPP group. Histological scores were lower in the F127@4-IPP group than in the blank F127 group, indicating reduced histopathological severity under the tested conditions. (Figure 8C). Immunohistochemical staining analysis demonstrated suppressed expression of inflammatory marker (MCP-1), apoptotic marker (Cleaved-Casp3), fibrotic marker (α-SMA), and migration-regulating marker (AQP1) in the F127@4-IPP group, compared to the F127 group at 1 week post-injury (Figures 8B and 8D). At 8 weeks post-injury, there was a reduction in protein expression of MCP-1 and α-SMA in the F127@4-IPP group (Figures 8B and D). The F127 group also showed cartilage glycosaminoglycan accumulation but not in the F127@4-IPP group (Figure 8G). Suppressed bone formation was further evidenced by the reduced OCN signal in the F127@4-IPP group (Figures 8E and 8F).

## Discussion

By reanalyzing a published single-cell dataset, this study characterized predicted communication patterns between mesenchymal and myeloid populations, identified increased MIF expression, and MIF signaling as candidate processes associated with inflammation and heterotopic ossification. Inhibition with 4-IPP was associated with reduced pathological remodeling in the experimental models. These findings suggest that tenocyte-lineage cells may participate in immune regulation during tendon injury and support further investigation of MIF-associated signaling as a potential therapeutic target.

Previous study has shown the tenocyte-immune cell interplay in an *in vitro* inflammation model.^43^ Our scRNA-seq analysis delineated a clear division of labor between tenocyte and myeloid lineages throughout the healing cascade. In the acute stage of injury (D1), tenocytes underwent a phenotypic switch from mature, matrix-producing cells (marked by Tnmd and Fmod) to inflammatory and proliferating subsets, characterized by the upregulation of *Mif*, *Cxcl1*, and *Ccl2*. This transcriptional shift coincides with the expansion of inflammatory macrophages and monocytes, which express high levels of Spp1 and Ptgs2. Pathway analysis confirms that this early phase is dominated by leukocyte chemotaxis and innate immune activation, providing a cellular basis for the acute pain and swelling observed in the clinical setting.^44^^,^^45^ By the mid-phase (D7), the narrative transitions from inflammation toward metabolic reprogramming. Tenocytes show pathway upregulation in wound healing, endothelial cell proliferation, and monosaccharide metabolism, while myeloid cells shift toward glycoprotein catabolism, macroautophagy, and lipid oxidation, reflecting a resolution phase. However, in the late phase (D28), the persistent expansion of fibrotic tenocytes, defined by Postn and Tgfb3, and dysregulated myeloid responses create a pathological niche, alongside the upregulation of pathways related to cartilage development and bone growth. Myeloid cell populations also show pathway upregulation in extracellular matrix assembly and response to peptidoglycan. These observations offer a potential explanation for the chronic degenerative changes seen in tendinopathy patients, such as collagen disorganization, fibrosis, and heterotopic bone formation.^46^^,^^47^ They also suggested the involvement of both tenocytes and myeloid cells in the series of degenerative events. The analysis here also addresses critical gaps in previous bulk transcriptomic studies by precisely characterizing the involvement of distinct cell subpopulations.^8^

Prevailing paradigms in tendon biology have long positioned macrophages and monocytes as the central arbiters of inflammation and tissue remodeling, while characterizing tenocytes merely as passive executors of collagen synthesis and matrix homeostasis.^48^^,^^49^ Our subclustering analysis challenges this hierarchical view, suggesting that tenocytes may function not only as a structural cell but also as active participants in immune regulation. This potential immunomodulatory capacity is further supported by our pathway analysis, which reveals that tenocyte-specific transcriptional programs may govern T cell lineage commitment at D1 and may regulate macrophage differentiation at D28. This temporal control over lymphoid and myeloid fate raises the possibility that tenocytes may function as the primary sentinels of tendon damage, potentially contributing to the inflammation associated with the healing process. Mechanistically, we identified that inflammatory, proliferating, and ECM-producing tenocyte subsets serve as the principal signal senders, with CellChat predicting potential communication with myeloid subpopulations through MIF-associated ligand-receptor pairs, including MIF-(CD74+CXCR4) and MIF-ACKR3. This computational prediction raises the possibility of a signaling relationship through which activated tenocytes may influence myeloid responses. Our findings align with recent studies highlighting that tendon and enthesis regeneration are regulated by broader inflammatory and metabolic networks involving macrophages, stem/progenitor cells, and stromal cells. For example, Gao et al. demonstrated that the P2X7R/NLRP3 inflammasome axis in macrophages suppresses enthesis regeneration through IL-1β-mediated inflammatory and docosatrienoic acid (DTA)-mediated metabolic cross-talk with stem cells.^50^ While that work identifies macrophages as primary drivers of both inflammatory and metabolic dysregulation, our study reveals that tenocytes themselves can initiate immune crosstalk through MIF signaling. This suggests that tenocyte-myeloid and macrophage-stem cell axes may operate sequentially or in parallel, with the MIF pathway representing an early, tenocyte-driven trigger that could prime or potentiate subsequent macrophage-mediated inflammasome activation and metabolic disruption. By bridging the gap between initial matrix disruption and systemic immune activation, these findings reposition tenocytes as key regulators in pathogenesis. These findings bridge the gap between initial matrix disruption and systemic immune activation, and align closely with emerging trends in research on mechanoresponsive tissues such as bone and cartilage, suggesting that mechanical load-bearing stromal cells may universally possess a core function in actively shaping the immune microenvironment.^51^^,^^52^

MIF is a cytokine with well-established roles in innate immunity, where it amplifies inflammatory responses by sustaining pro-inflammatory cytokine production, inhibiting glucocorticoid action, and promoting leukocyte recruitment.^53^^,^^54^ In tendinopathy, prior studies have suggested its upregulation and promoting effects during tendon heterotopic ossification.^55^ Our findings extend these observations by demonstrating that inflammatory and proliferating tenocyte subsets are computationally predicted to be the dominant signal senders, with predicted engagement of myeloid cells via the receptors CD74/CXCR4 and ACKR3 at D1, coinciding with the acute inflammatory stage. These predictions were derived from CellChat analysis and require orthogonal validation—such as spatial protein co-localization or receptor blockade experiments. Furthermore, clinical microarray data reveal that patients with high *MIF* expression exhibited upregulation of stress, inflammatory, fibrotic, and ossification pathways, collectively demonstrating an association between elevated *MIF* expression and molecular programs related to inflammation, fibrosis, and ossification. This was further supported by exogenous MIF injection in mouse Achilles tendon, which recapitulated the features of tendinopathy, not only histological changes including inflammatory infiltrate, vascularization, collagen disorganization, but also the upregulation of key pathological markers. These observations are consistent with the reported functions of MIF in other musculoskeletal disorders, including rheumatoid arthritis, intervertebral disc degeneration, and osteoarthritis.^56^^,^^57^^,^^58^ These findings suggest that MIF-associated signaling may contribute to pathological remodeling. However, its causal role in the transition from acute injury to persistent tendon pathology remains to be established.

We next evaluated pharmacological MIF inhibition using 4-IPP.^42^ *In vitro*, 4-IPP attenuated IL-1β induced inflammatory, apoptotic, chondrogenic, and osteogenic changes in TDSCs under the tested conditions. In the mouse Achilles tendon injury model, the F127@4-IPP formulation was associated with reduced inflammatory infiltration, histopathological severity, and osteogenic or chondrogenic changes. These findings provide preliminary pharmacological evidence supporting further evaluation of MIF-associated processes and 4-IPP in tendon injury. Additional studies are required to establish target specificity, functional benefit, *in vivo* retention, and biosafety.

### Limitations of the study

Several limitations of this study warrant consideration. Firstly, while we identified MIF as a signaling pathway in the acute stage of pathogenesis and the upstream mechanism that triggers its upregulation in tenocytes following injury still remains unclear. Secondly, we did not perform validation in MIF genetic knockout animal models, which would be necessary to establish the causal role of the MIF pathway. Thirdly, we did not conduct *in vivo* functional studies to examine the therapeutic effects of 4-IPP on biomechanical strength or locomotor recovery, which is the ultimate goal for healing. Moreover, we acknowledge that TDSCs are progenitor cells and RAW264.7 is an immortalized cell line, which differ from primary tenocytes and macrophages and may not fully reflect the physiological mechanisms. Finally, the puncture-induced injury model, while valuable for delineating acute inflammatory dynamics, represents a traumatic condition that may not fully recapitulate the chronic, low-grade degenerative microenvironment of overuse tendinopathy or Achilles tendon rupture—two scenarios that are most prevalent in the clinical setting. Future studies should include tenocyte-specific MIF knockout models to confirm the role of tenocyte-derived MIF, alongside receptor-specific blockade (CD74, CXCR4, and ACKR3), primary tenocyte, and primary macrophage validation to define the signaling axis. Additional *in vivo* controls, such as free 4-IPP and solvent vehicle groups, are needed to distinguish sustained release effects from direct drug action. Anatomically matched clinical samples and functional outcome measures including biomechanical testing, gait analysis, and quantitative HO assessment will further validate the translational relevance and functional impact of MIF inhibition in tendinopathy. Additionally, studies involving human subjects are warranted to provide a more complete understanding of MIF’s role in clinical tendinopathy.

### Conclusion

Together, our findings characterize the dynamic cellular and signaling landscape of tendon healing after puncture-induced injury and identify MIF-associated signaling as a candidate pathway linked to tendon pathological remodeling through mesenchymal cell-myeloid cell crosstalk. F127@4-IPP attenuated histopathological and molecular changes in the puncture-induced mouse tendon injury model, supporting further evaluation of MIF inhibition as a potential therapeutic strategy.

## Resource availability

### Lead contact

Further information and requests for resources and reagents should be directed to and will be fulfilled by the lead contact, Zebin Ma (mazebin0300@163.com).

### Materials availability

This study did not generate new unique reagents.

### Data and code availability

• Data: This paper analyzes existing, publicly available data, accessible at GEO: GSE288444^8^ and GSE26051.^41^
• Code: This study did not generate any original code.
• Any additional information required to reanalyze the data reported in this paper is available from the lead contact upon request.

## Acknowledgments

The study was supported by the 10.13039/501100002858China Postdoctoral Science Foundation under Grant No. 2025M782137.

## Author contributions

Conceptualization, Z.M.; data curation, C.H.K., X.X., P.H., X.L., and G.C.; formal analysis, C.H.K., X.X., P.H., X.L., and G.C.; funding acquisition, Z.M.; investigation, C.H.K., X.X., P.H., X.L., and G.C.; methodology, C.H.K. and X.X.; project administration, C.H.K. and X.X.; resources, Z.M.; supervision, Z.M.; validation, C.H.K., X.X., P.H., X.L., and G.C.; writing – original draft, C.H.K. and X.X.; writing – review and editing, C.H.K., X.X., and Z.M.

## Declaration of interests

The authors declare no competing interests.

## STAR★Methods

### Key resources table

**Table:** 

REAGENT or RESOURCE	SOURCE	IDENTIFIER
**Antibodies**		

Anti-MIF antibody	Abcam	Cat#ab36146; RRID:AB_776371
Mouse Decorin Antibody	Invitrogen	Cat#MAB1060; RRID:AB_2090385
Anti-MCP1 antibody	Abcam	Cat#ab25124; RRID:AB_448636
Alpha smooth muscle actin Polyclonal antibody	Proteintech	Cat#14395-1-AP; RRID:AB_2223009
CD64 Recombinant Rabbit Monoclonal Antibody	Invitrogen	Cat#MA5-29706; RRID:AB_2785530
Anti-CD68 antibody	Abcam	Cat#ab125212; RRID:AB_10975465
Cleaved Caspase-3 antibody	Cell Signaling Technology	Cat#9661; RRID:AB_2341188
AQP1 Polyclonal antibody	Proteintech	Cat#20333-1-AP; RRID:AB_10666159
Anti-Osteocalcin antibody	Abcam	Cat#ab93876; RRID:AB_10675660
Anti-Rabbit IgG (H+L), HRP	Invitrogen	Cat#31460; RRID:AB_228341
Anti-Rabbit IgG (H+L), Alexa Fluor™ 488	Invitrogen	Cat#A-11008; RRID:AB_143165
Anti-Rat IgG (H+L), Alexa Fluor™ 647	Invitrogen	Cat#A-21247; RRID:AB_141778
Anti-Goat IgG (H+L), Alexa Fluor™ 488	Invitrogen	Cat#A-11055; RRID:AB_2534102

**Biological samples**		

Mouse tendon	This paper	N/A
Human hamstring tendon	This paper	N/A
Human rotator cuff tendon	This paper	N/A

**Chemicals, peptides, and recombinant proteins**		

Mouse recombinant MIF protein	MedChemExpress	Cat#HY-P700517
4-IPP	MedChemExpress	Cat#HY-110063
Mouse IL-1 beta Recombinant Protein	Gibco	Cat#211-11B-50UG
Polyether F127 Diacrylate	Engineering For Life	Cat#EFL-F127DA-001
ProLong™ Diamond Antifade Mountant with DAPI	ThermoFisher	Cat#P36962
DMEM, high glucose	Gibco	Cat#11965092
MEM α, nucleosides	Gibco	Cat#12571063
Penicillin-Streptomycin	Gibco	Cat#15140122
Fetal Bovine Serum	Gibco	Cat#A5256701
Collagenase type I	Gibco	Cat#17100017
Trypsin-EDTA (0.25%), phenol red	Gibco	Cat#25200056
Alizarin Red S	Sigma	Cat#A5533
Lipofectamine 3000	Thermofisher	Cat#L3000001
Osteogenic medium	OriCell	Cat#MUXTA-90021
FastPure RNA kit	Vazyme	Cat#RC101
HiScript III All-in-one RT SuperMix	Vazyme	Cat#R323
TB Green Premix Ex Taq	Takara	Cat#RR820A

**Critical commercial assays**		

CCK-8 assay kit	Abcam	Cat#ab228554

**Deposited data**		

scRNA-seq raw data and analyzed data	Huang et al.^8^	GSE288444
Microarray data	SA et al.^41^	GSE26051

**Experimental models: Cell lines**		

RAW264.7	ATCC	TIB-71

**Experimental models: Organisms/strains**		

Mus musculus C57BL/6N strain	Charles river	https://www.vitalriver.com/rm-find-model/213-c57bl-6n.html

**Oligonucleotides**		

Primers for RT-qPCR, see Table S1	This paper	N/A
Mif Mouse siRNA Oligo	Origene	SR401629
Trilencer-27 Universal Scrambled Negative Control siRNA	Origene	SR30004

**Software and algorithms**		

R	The R Foundation for Statistical Computing	https://www.r-project.org/
Seurat	Satija Lab	https://satijalab.org/seurat
Harmony	Korsunsky et al.^59^	https://github.com/immunogenomics/harmony
DoubletFinder	McGinnis et al.^60^	https://github.com/chris-mcginnis-ucsf/DoubletFinder
CellChat	Jin et al.^61^	https://github.com/sqjin/CellChat
ClusterProfiler	Bioconductor	https://bioconductor.org/
ggplot2	CRAN	https://cran.r-project.org/
GraphPad Prism v8	GraphPad Software	https://www.graphpad.com/
ImageJ	NIH	https://imagej.nih.gov/

### Experimental model and study participant details

#### Ethical statements

All human clinical studies were approved by the Ethics Committee of the First Affiliated Hospital of Shantou University Medical College (approval number: B-2023-052), with written informed consent obtained from all participants. All animal experiments were approved by the Animal Ethics Committee of Shantou University Medical College (approval number: SUMCSY2025-030) and performed in accordance with institutional guidelines.

#### Animal studies

Eight- to ten-week-old male C57BL/6N mice were used for all experiments. All mice were housed with *ad libitum* access to standard chow and tap water under pathogen-free conditions, including a 12-h light/ 12-h dark cycle, 30%–70% relative humidity, and a temperature of 20°C–23°C. The puncture-induced tendon injury model was constructed based on a previous method from Huang et al.^8^ Mice were anesthetized by intraperitoneal injection of ketamine/xylazine. After cleaning and disinfection of the surgical area, a longitudinal incision (∼5 mm) was made to expose the Achilles tendon on the right hindlimb while ophthalmic forceps were positioned beneath the tendon to provide support for the needling procedures. The tendon was then punctured using the needles, and the phenotypic and osteogenic changes in the model were confirmed by histological staining (Figure S2). The incision was closed with 6-0 sutures. Mice received postoperative analgesia and were monitored during recovery. For recombinant MIF intervention, recombinant mouse MIF protein at a dose of 25 μg/kg was injected around the Achilles tendon three times per week. For the preparation of F127@4-IPP, lithium phenyl-2,4,6-trimethylbenzoyl phosphinate (LAP; Engineering for Life) photoinitiator was dissolved in a 45 °C water bath for standby use. Hydrophobic 4-IPP (Sigma) was dissolved in absolute ethanol and combined with LAP solution and Polyether F127 diacrylate powder (F127DA; Engineering for Life). The mixture was stirred at 2°C–8°C under dark conditions to volatilize ethanol and yield the F127@4-IPP hydrogel product. For the intervention of F127@4-IPP hydrogel, the mice were randomly assigned to one of three groups including Sham group, injury with F127 hydrogel, injury with F127@4-IPP hydrogel. At the time of injury, 20 μL of the mixture of F127DA, LAP, and 4-IPP at a dose of 1 mg/kg was administered to the injured tendon site. F127DA underwent thermally induced gelation at body temperature, followed by 405 nm light irradiation for 10 seconds to complete photocrosslinking.

### Method details

#### scRNA-seq data processing

Raw data for the four individual groups of the scRNA-seq were downloaded from the GEO database (GSE288444) and the gene expression data were processed with the Seurat package (5.4.0) in R software (v4.4.1). Low-quality cells were filtered, identified as having more than 20% mitochondrial genes and fewer than 300 feature genes (Figure S1). Doublets were identified and removed using the DoubletFinder package (v2.0.4).^60^ After filtering, SCTransform normalization was performed with the top 2000 features while the variables of “nCount_RNA” and “percent.mito” were regressed out during scaling. Batch correction for individual samples was performed using Harmony (v1.2.4).^59^ Nearest-neighbour graph construction, cluster determination and nonlinear dimensionality reduction were performed using the FindNeighbors, FindClusters and RunUMAP functions, respectively.

#### Differential gene expression and pathway analysis

Differential gene expression was calculated using the FindAllMarkers function with the Wilcoxon rank-sum test. Volcano plots were generated using the ggplot2 package (v4.0.1) to visualize the differentially expressed genes (DEGs). Gene ontology analysis was employed to identify the pathway enrichment using the ClusterProfiler package on the DEGs (v4.14.6). Detailed results of DEGs and GO pathway enrichment for main clusters and subclusters are provided in Tables S2 and S7.

#### Cell-cell communication analysis

Cell-cell communication analysis was conducted using the CellChat package (v2.2.0). A CellChat object was created from the Seurat object, and CellChatDB.mouse database was used to identify the signaling pathways. The aggregated cell–cell communication network was calculated using the aggregateNet function, and signaling from each population was visualized. Incoming and outgoing signals of different populations were calculated using the function netAnalysis_signalingRole_heatmap. Ligand-receptor pairs and information flow were identified, and communication strengths were calculated using the computeCommunProb function.

#### Characterization of F127@4-IPP hydrogel

The microstructure of lyophilized hydrogels was examined by scanning electron microscopy (SEM; Quanta-400, FEI, USA) at an accelerating voltage of 10 kV, with samples sputter-coated with gold prior to imaging. *In vitro* drug release profiles were assessed using the dialysis bag method in PBS (pH 7.4) at 37 °C with constant shaking at 100 rpm; at predetermined intervals (0, 1, 3, 5, 7, and 14 days), 10 μL of release medium was sampled and replaced with fresh PBS, and the cumulative release of 4-IPP was quantified by UV-Vis spectrophotometry at 260 nm (*n* = 3 per group).^62^ Swelling kinetics were evaluated gravimetrically in PBS at 37 °C; lyophilized hydrogels of known dry weight (W_0_) were immersed in PBS and weighed (W_t_) at designated time points (0, 0.5, 1, 3, 5, and 24 h) after surface water removal, with the swelling ratio calculated as (W_t_ − W_0_)/W_0_ × 100% (*n* = 3 per group).^63^ To evaluate drug loading capacity, 4-IPP was incorporated into F127DA micelles at theoretical feeding ratios of 0.5% and 2.5% (w/w); The F127DA@4-IPP precursor solution was centrifuged at 14,000 rpm for 30 min at 4°C. The pellet containing non-encapsulated 4-IPP was redissolved in absolute ethanol and quantified by UV–Vis spectrophotometry at 260 nm using a standard calibration curve. Encapsulation efficiency was calculated as: Encapsulation efficiency was calculated by subtracting the non-encapsulated 4-IPP mass recovered from the pellet from the initial 4-IPP mass, dividing the result by the initial 4-IPP mass, and multiplying by 100%. *In vitro* degradation was assessed by incubating hydrogel samples (*n* = 5 per group) of known initial weight in PBS (pH 7.4) at 37 °C; at predetermined time points, samples were removed, lyophilized, and weighed, with the weight loss calculated as (W_0_ − W_t_)/W_0_ × 100%.

#### Histology, immunohistochemistry (IHC) and immunofluorescence (IF)

Human hamstring, rotator cuff and mouse Achilles tendon were fixed in 10% neutral buffered formalin at room temperature for 24 h and then dehydrated through a graded ethanol series (70–100%), cleared in xylene and embedded in paraffin. The blocks were sectioned at a thickness of 5 μm using microtome and stained with Hematoxylin and Eosin (H&E) and Safranin O/Fast Green (SOFG) following standard protocol.^64^ Semi-quantitative analysis of H&E staining results was conducted according to a published protocol.^64^ For IF, tissue sections were deparaffinized in xylene and rehydrated in decreasing concentration of ethanol to PBS. Then antigen retrieval was performed with 10mM sodium citrate buffer (pH6.0) at 95°C for 30 minutes. Sections were blocked in blocking solution for half an hour, prepared with 3% bovine serum albumin (BSA) in PBST. The sections were then incubated with primary antibodies at various dilutions in blocking solution at 4°C overnight. After washing with PBST three times, the sections were then incubated with secondary antibodies at room temperature for 45 minutes . After washing with PBST three times, the sections were mounted with DAPI mounting medium (Thermo Fisher Scientific). For IHC, after incubation with primary antibodies followed by PBST washing, the sections were incubated with horseradish-peroxidase-conjugated secondary antibodies for 1 hour, and positive signals were visualized with a DAB kit (Thermo Fisher Scientific). Images were captured under a fluorescence microscope (Leica Microsystems, Wetzlar, Germany).

#### TDSCs isolation and culture

Human hamstring, rotator cuff samples and mice Achilles tendon were surgically excised and rinsed twice with PBS containing 1% penicillin/streptomycin (Thermo Fisher Scientific). The samples were then incubated in 0.25% Trypsin-EDTA (Gibco) at 37°C for 5 minutes, followed by washing with PBS. The samples were then fragmented to approximately 1mm^3^ with ophthalmic scissors and incubated in 3 mg/mL collagenase type I (Thermo Fisher Scientific) dissolved in Minimum Essential Medium α (αMEM; Gibco) at 37°C for one hour. The digested tissues were passed through a 70 μm cell strainer (Corning), and the resulting single cell suspension was centrifuged by 1000 rpm for 5 minutes. The supernatant was then discarded and the cell pellet was resuspended in αMEM supplemented with 10% FBS and 1% P/S. Cells were seeded in 500 cells/cm^2^ in cell culture dishes and incubated at 37°C with 5% CO2 for 14 days. Colonies were trypsinized with 0.25% Trypsin-EDTA (Gibco) at 37°C for 5 minutes and passaged to new cell culture dishes for expansion and designated as passage 1. All experiments were performed with cells from passages 3 to 7 unless otherwise specified.

#### RAW 264.7 cell line and culture

The murine macrophage-like RAW 264.7 cell line was purchased from the American Type Culture Collection (ATCC, TIB-71). Cells were cultured in high-glucose DMEM (Thermo Fisher Scientific) supplemented with 10% fetal bovine serum (Thermo Fisher Scientific) and 1% penicillin-streptomycin (Thermo Fisher Scientific), and maintained at 37°C in a humidified incubator with 5% CO_2_.

#### Viability assay

TDSCs were seeded at 3000 cells/well in a 96-well cell culture plate and incubated at 37°C with 5% CO2 for overnight. The cells were then treated with various doses of 4-IPP and IL-1β (10ng/mL; Gibco) for 24 h. After that, cell viability was assessed using the CCK-8 assay kit (Abcam) according to the manufacturer’s instructions. Briefly, TDSCs were incubated with 10% CCK-8 reagent in complete medium for 2 h. Absorbance was measured at 450 nm using a plate reader.

#### Osteogenic differentiation and Alizarin Red S staining

TDSCs were seeded at 100,000 cells/well in a 6-well cell culture plate and incubated at 37°C with 5% CO2 until 90% confluency was reached. The culture medium was replaced with complete osteogenic induction medium (OriCell) for continuous culture with or without 4-IPP (10μM) until day 21, with renewal of the medium every 2–3 days. TDSCs were then rinsed with PBS, treated with 4% paraformaldehyde for 30 minutes and incubated with 1% Alizarin Red S (Sigma-Aldrich) for 30 minutes. Three repeated washes with PBS were required to eliminate the excess dye.

#### siRNA-mediated MIF gene knockdown

Mif knockdown was performed using Mif-targeting siRNA (OriGene) or a scramble control. TDSCs were seeded at 3 × 10^4^ cells per well in 12-well plates overnight and transfected with 10 nM siRNA using Lipofectamine 3000 (Thermo Fisher Scientific) in Opti-MEM (Gibco). After transfection, cells were subjected to osteogenic induction or harvested for RNA analysis.

#### RNA extraction, cDNA synthesis and qRT-PCR

RNA was extracted from TDSCs and RAW 264.7 cells using the FastPure Cell/Tissue Total RNA Isolation Kit (Vazyme). A total of 1000 ng RNA was used for cDNA synthesis using the HiScript III All-in-one RT SuperMix (Vazyme). 20 μL reactions were prepared for each well in 384-well plate (Applied Biosystems) using TB Green Premix Ex Taq (Takara). qRT-PCR was performed using QuantStudio7 Pro 384 (Applied Biosystems) following manufacturer’s protocol. The primers used were listed in Table S1. The gene expression fold change was calculated using the 2ˆ^-ΔΔCT^ formula while *Actb* was used as the housekeeping gene.

### Quantification and statistical analysis

To account for multiple comparisons in single-cell RNA sequencing analyses, P-values were adjusted using the Benjamini–Hochberg (BH) procedure to control the false discovery rate (FDR). Specifically, FDR correction was applied to differential gene expression analysis using Seurat FindAllMarkers function, functional enrichment analyses using clusterProfiler function, and ligand-receptor interaction significance using CellChat function. All adjusted P-values reported in the Results section represent FDR-adjusted values. All experiments were performed independently at least 3 times to ensure reproducibility. Statistical analyses were carried out using R package and GraphPad Prism software (v9.3). Differences between groups were analyzed by unpaired Student’s two-tailed *t* test with Welch’s correction, one-way ANOVA with Tukey test, or Mann-Whitney test. *p* < 0.05 was used to identify statistical significance. Statistical plots were made using the ggplot2 package and GraphPad Prism software. Data are presented as mean ± standard deviation (SD). The statistical details of each experiment can be found in the figure legends and Table S8.

## Supplemental information

Document S1. Figures S1–S4 and Table S1

Table S2. Top DEGs of main clusters

Table S3. Top GO pathway enrichment of different clusters

Table S4. Top DEGs of mesenchymal cell subpopulations

Table S5. Top GO pathway enrichment of mesenchymal cell subpopulations

Table S6. Top DEGs of myeloid cell subpopulations

Table S7. Top GO pathway enrichment of myeloid cell subpopulations

Table S8. Statistical analysis output of each experiment
