# Supplementary material for: Single-cell transcriptomics reveals that tenocyte-derived MIF contributes to tendon injury pathogenesis
Source: iScience. 2026 Sep 1;29(9):117411. doi: 10.1016/j.isci.2026.117411 (PMC13571148; doi:10.1016/j.isci.2026.117411)
Supplement: Supplementary file 1 [file mmc1.pdf]

iScience, Volume *29*

Supplemental information

Single-cell transcriptomics reveals that tenocyte-derived MIF contributes to tendon injury pathogenesis

Cheuk Hin Kot, Xiaojie Xu, Peifeng Hong, Xu Liang, Gerun Chen, and Zebin Ma

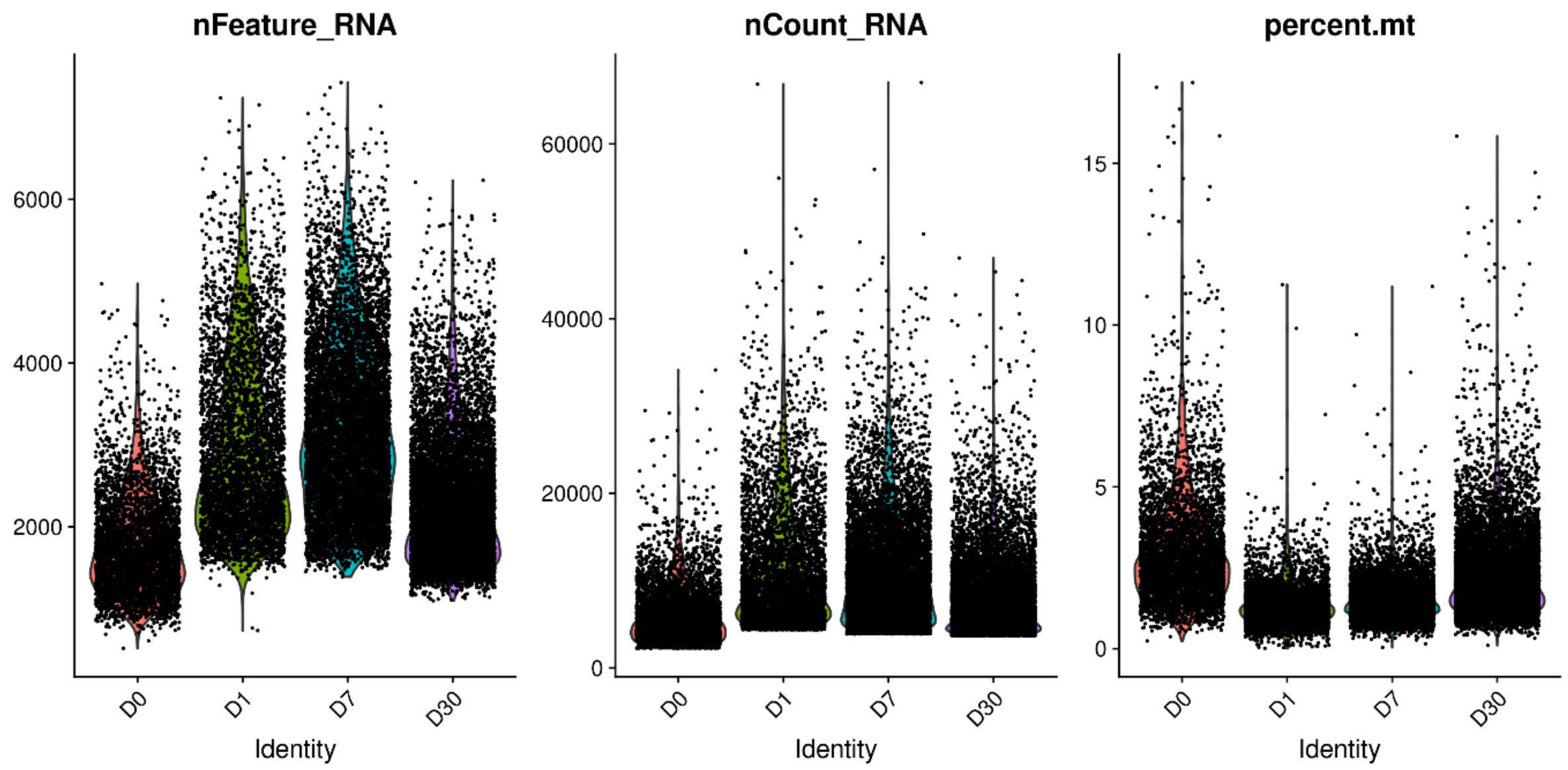

Figure S1. ScRNA-seq quality control information.

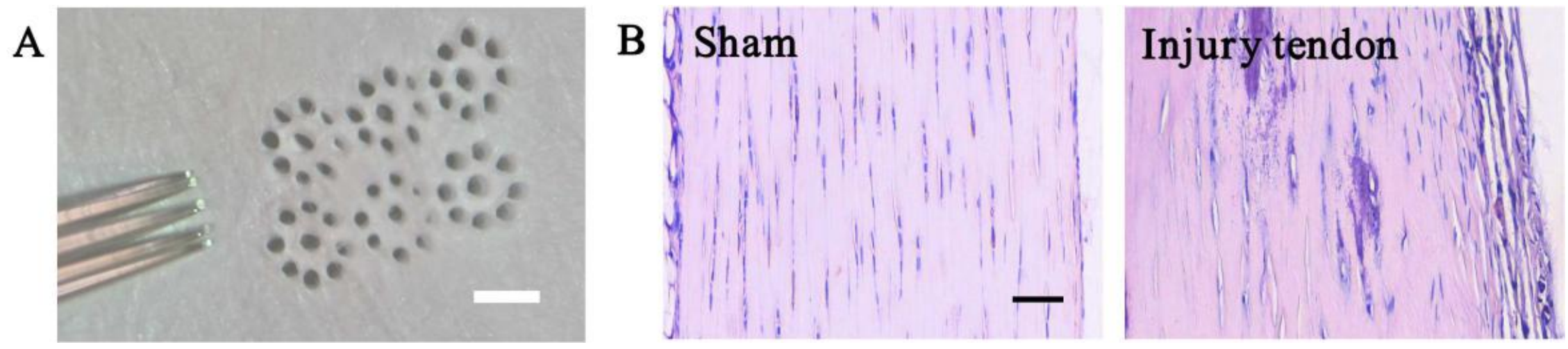

Figure S2. Needle puncture tendon injury model. (A) Macroscopic photograph of the multi-pronged needle utilized to establish tendon puncture injury. Scale bar = 0.5 mm. (B) Representative histological staining of tendon tissues from the sham control group and injured tendons at 8 weeks post needle puncture. Scale bar = 100 μm.

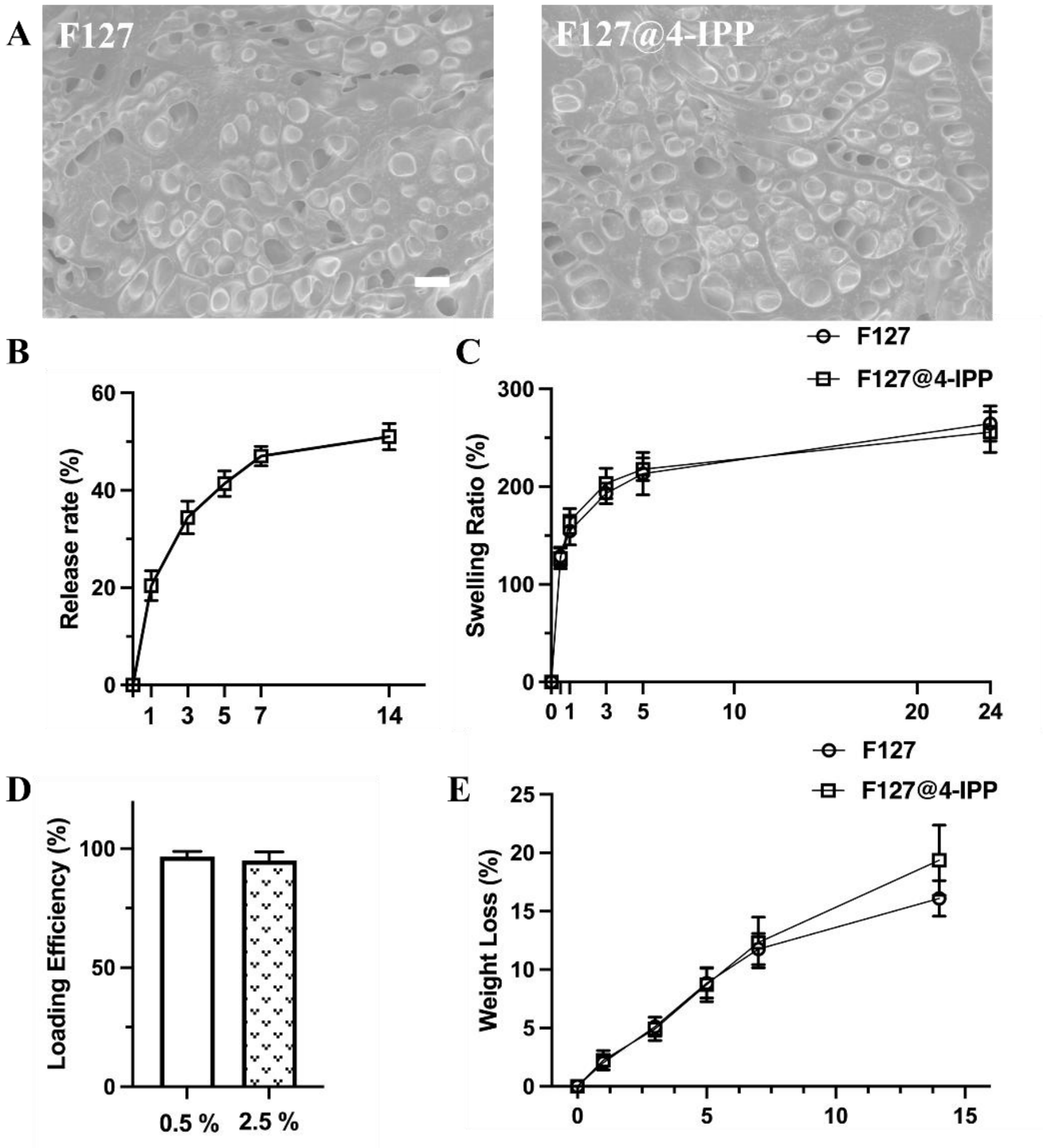

Figure S3. Characterization of 4-IPP-loaded photo-crosslinked F127DA hydrogel. (A) Representative SEM images showing the microstructure of blank F127DA and 4-IPP-loaded F127DA hydrogels. Scale bar, 10 μm. (B) The 14-day cumulative release profile of 4-IPP from F127DA hydrogel in pH 7.4 PBS at 37 °C. Swelling ratios of blank and 4-IPP-loaded F127DA hydrogels in PBS at 37°C over 24 h (n = 3). (D) Encapsulation efficiency of 4-IPP-loaded F127DA hydrogels at a theoretical feeding ratio of 0.5 and 2.5% (w/w). (E) Weight loss of blank and 4-IPP-loaded F127DA hydrogels during incubation in PBS at 37°C for 14 days (n = 5).

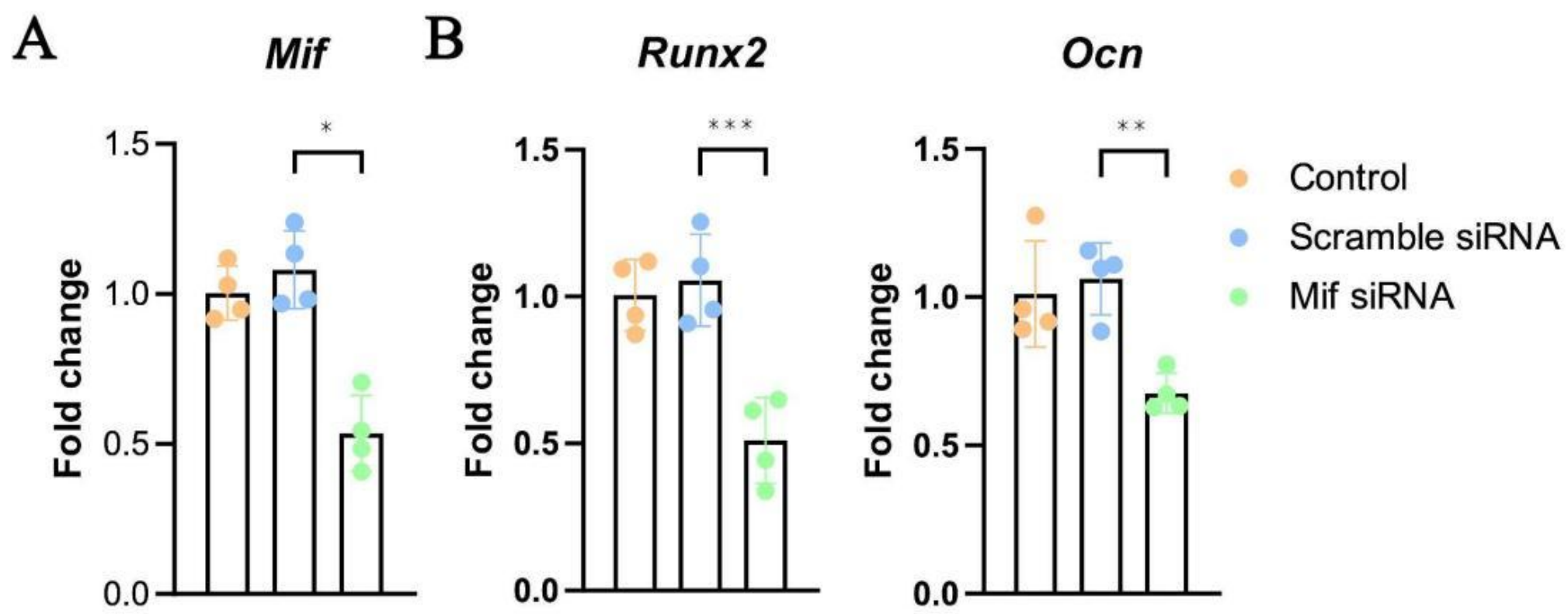

Figure S4. siRNA-mediated Mif knockdown reduces osteogenic-marker expression in TDSCs. (A) Mif mRNA expression after transfection with scramble or Mif-targeting siRNA (n = 4 per group). (B) Runx2 and Ocn mRNA expression after 21 days of osteogenic induction (n = 4 per group). Statistical significance was assessed by one-way ANOVA followed by Tukey's test. Data are mean ± SD; *$p < 0.05$, **$p < 0.01$, ***$p < 0.001$.

Supplementary Table

Table S1. Primers for qRT-PCR

Gene name	Forward primer	Reverse primer
Actb	TAAAACGCAGCTCAGTAACAGTCC	CTCCTGAGCGCAAGTACTCTGTG
Mif	CCGCACAGTACATCGC	GATGTAGACCCGGTCCG
Il6	CTGATGCTGGTGACAACCAC	GCCACTCCTTCTGTGACTCC
Tnf-α	CCCTCACACTCAGATCATCTTCT	GCTACGACGTGGGCTACAG
Bax	CTCGCTTCGGCAGCACA	AACGCTTCACGAATTTGCGT
Bad	CATCATGGAGGCGCAG	GCGAGGAAGTCCCTTGAAG
Acan	GTGGAGCCGTGTTTCCAAG	AGATGCTGTTGACTCGAACCT
Comp	GGTCTTGCGTGTGTGC	GCAGTTGTCCTTGCGAC
Runx2	GGCAAGATGAGCGACGTGAG	ATCTGACTCTGTCCTTGTGG
Ocn	GAACAGACAAGTCCCACACAGC	TCAGCAGAGTGAGCAGAAAGAT
Inos	GGAGTGACGGCAAACATGACT	TCGATGCACAACTGGGTGAAC
Il1β	CAACCAACAAGTGATATTCTCCATG	GATCCACACTCTCCAGCTGCA
